# Effects of Foliar Selenite on the Nutrient Components of Turnip (*Brassica rapa* var. *rapa* Linn.)

**DOI:** 10.3389/fchem.2018.00042

**Published:** 2018-03-02

**Authors:** Xiong Li, Boqun Li, Yongping Yang

**Affiliations:** ^1^Key Laboratory for Plant Diversity and Biogeography of East Asia, Kunming Institute of Botany (CAS), Kunming, China; ^2^China Germplasm Bank of Wild Species, Kunming Institute of Botany (CAS), Kunming, China

**Keywords:** selenium, biofortification, turnip, nutritional value, human health

## Abstract

We administered foliar applications of 50, 100, and 200 mg L^−1^ selenium (Se, selenite) on turnip (*Brassica rapa* var. *rapa* Linn.) and detected the changes in the main nutrient components in fleshy roots. Results showed that the foliar application of Se (IV) significantly increased the Se content in turnip, and Se (IV) positively affected the uptake of several mineral elements, including magnesium, phosphorus, iron, zinc, manganese, and copper. Se (IV) treatments also improved the synthesis of protein and multiple amino acids instead of crude fat and total carbohydrate in turnip, indicating that the foliar application of Se (IV) could enhance Se biofortification in turnip and promote its nutritional value. We recommended 50–100 mg L^−1^ Se treatment for foliar application on turnip based on the daily intake of Se for adults (96–139 μg person^−1^ day^−1^) and its favorable effects on the nutrient components of turnip.

## Introduction

Selenium (Se) is an essential micronutrient for humans (White and Brown, 2010). Se deficiency can weaken the immune system and increase the risks of several diseases, including hypothyroidism, cardiovascular disease, or various cancers (Fairweather-Tait et al., 2011; Rayman, 2012; White, 2016). Excessive dietary Se intake can also cause selenosis in humans (Fairweather-Tait et al., 2011; Rayman, 2012; Sperotto et al., 2014), and its symptoms are similar to those caused by heavy metals (White, 2016). As such, the World Health Organization (WHO) has recommended the dietary allowance of ~55–200 μg Se day^−1^ for adults (Wu et al., 2015), and the Institute of Medicine (USA) has suggested a tolerable upper intake of 400 μg Se day^−1^ for adults (White, 2016). Se supplementation greatly depends on the production of crops, vegetables, or edible mushrooms on soils with substantial Se content or phytoavailability (Broadley et al., 2006; Chilimba et al., 2011; Joy et al., 2015; Dogan et al., 2016). Se accumulation in tissues can be used as a basis for the classification of angiosperm species into three ecological types: non-accumulator, Se-indicator, and Se-accumulator species (White et al., 2007; White, 2016). Some studies have focused on the potential of plants, such as Se-accumulator species, for the phytoremediation of Se-contaminated soils (Banuelos and Dhillon, 2011; Wu et al., 2015). However, much interest has been directed toward developing Se-biofortified agricultural products because of severe Se deficiency worldwide (Wu et al., 2015). For example, inorganic Se fertilizers have been applied to effectively increase Se contents in diets and thus improve the Se status and health of humans (White and Broadley, 2009; Alfthan et al., 2015).

For plants, Se is a beneficial element because it can stimulate plant growth and enhance plant tolerance or resistance to abiotic or biotic stress (Quinn et al., 2007; Pilon-Smits et al., 2009; Feng et al., 2013). However, Se toxicity in plants has also been reported (Fu et al., 2011; Mao et al., 2011; Longchamp et al., 2015). A low Se concentration is considered advantageous for plant growth and development, whereas a high Se concentration can be toxic to plants (Fu et al., 2011; Mao et al., 2011; Gupta and Gupta, 2017). These effects may be attributed to changes in biochemical and metabolic processes caused by Se. For example, Se can mediate reactive oxygen species metabolism and trace element uptake in plants (Gupta and Gupta, 2017). Appropriate Se concentrations also improve the nutrient components, including mineral elements, polysaccharides, proteins, amino acids, and vitamin C, of some vegetables or edible mushrooms (Shang et al., 1998; Du et al., 2004; Zhao et al., 2004; Wang et al., 2005, 2006). Se enhances the synthesis of glucosinolates, which are important secondary metabolites found mainly in cruciferous plants (Sams et al., 2011; Malagoli et al., 2015; Schiavon et al., 2016). These reports fully support the conclusion that Se assimilation affects sulfur (S) and nitrogen (N) metabolic pathways in plants (Malagoli et al., 2015).

Turnip (*Brassica rapa* var. *rapa* Linn.), a cruciferous biennial plant, has been widely cultivated as a long-term vegetable or fodder in Europe, America, and Asia. It is rich in vitamin C, riboflavin, dietary fiber, and various mineral elements but is low in calories (Parveen et al., 2015; Ma et al., 2016). This biennial plant also contains antioxidants and can reduce the risk of high blood pressure, diabetes, and different cancers (Parveen et al., 2015). In China, turnip is mainly cultivated in the Qinghai–Tibet Plateau and its surrounding areas. However, these areas are exposed to severe Se deficiency. In a previous study, we analyzed the absorption and translocation characteristics of selenite and selenate in turnip by soil addition or foliar spraying (Li et al., 2018). We reported that turnip may be a potential Se-indicator species, and Se (IV) should be mainly selected as artificial Se fertilizer for turnips (Li et al., 2018). These results provided preliminary information on Se intake from turnip for local people, but the risk of Se biofortification, such as the assimilation of Se in plants and the effects of Se on turnip quality, is still unknown in turnips. In the present study, we examined the changes in nutrient components in turnips treated with Se (IV) concentration gradient via foliar application. We assumed that suitable Se concentrations can promote the nutrition level of turnip. Our results would further improve the understanding of the potential and value of Se biofortification in turnips.

## Materials and methods

### Plant cultivation and treatment

Turnip seeds from Ninglang County of China (Landrace No. KTRG-B54) were germinated and grown in a natural environment. After 25 days, seedlings with consistent growth were neatly transplanted into uniform flowerpots (*d* = 18.5 cm, *h* = 17.5 cm) with equal amounts of Se-free mucky soil (one seedling in each pot). The soil condition is shown in Table 1. The pots were divided into four groups, and each group contained three pots. The different groups of plants were supplied with 0, 50, 100, and 200 mg L^−1^ Se (IV) solutions by foliar application 25 and 40 days after the plants were transplanted. For each treatment, the Se (IV) solutions were sprayed until water droplets formed on the surface of the leaves. The pots were then placed in a transparent plastic shed with appropriate watering. The plants in each treatment were harvested 60 days after transplantation for subsequent measurement.

**Table 1:** Parameters of the soil used in the experiment.

**Parameter**	**Unit**	**Value**
pH	/	6.80
Organic matter	g kg^−1^ DW	214
Exchangeable Ca	cmol kg^−1^ DW	20.3
Exchangeable Mg	cmol kg^−1^ DW	2.24
Exchangeable K	cmol kg^−1^ DW	0.44
Available Zn	mg kg^−1^ DW	10.1
Available Fe	mg kg^−1^ DW	25.7
Available Mn	mg kg^−1^ DW	34.8
Available Cu	mg kg^−1^ DW	0.78
Total Se	mg kg^−1^ DW	0.08

### Sample preparation and biomass measurement

The fleshy roots of plants were harvested and washed with distilled water. The fresh weights of the root samples were measured, and the samples were subsequently dried in an oven at 80°C for 48 h to determine their dry biomasses. The conversion factors necessary to convert the fresh weight to the dry weight (DW) of the experimental plants were calculated for each treatment. The dry samples were then used for the following measurements. Three biological replicates were prepared for each treatment.

### Mineral analysis

Calcium (Ca), potassium (K), magnesium (Mg), and phosphorus (P) contents were determined in accordance with the method described by Li et al. (2016) by using an inductively-coupled plasma spectrometer (ICP-OES Optima 8000, Perkin Elmer, USA). The contents of Se, copper (Cu), iron (Fe), manganese (Mn), and zinc (Zn) were determined in accordance with the method of Li et al. (2017) by using an inductively-coupled plasma mass spectrometer (ICP-MS, Thermo Fisher Scientific, USA). The daily intake of Se for adults was calculated on the basis of the Se concentrations as follows: daily intake of Se = Se concentrations in plants (μg kg^−1^ DW) × conversion factor × daily intake of vegetables. The conversion factors 0.120, 0.116, 0.104, and 0.093 were used to convert the fresh weight to DW of the samples Se0, Se50, Se100, and Se200 in this study, respectively. The average daily vegetable intake for adults is 0.345 kg person^−1^ day^−1^ (Parveen et al., 2015).

### Macronutrient analysis

The crude protein content (N × 4.38) was determined using Kjeldahl method (Du et al., 2004; Liu et al., 2016). The crude fat content was determined through Soxhlet extraction with petroleum ether as a solvent (Du et al., 2004; Liu et al., 2016). The total carbohydrate content was measured using a phenol–sulphuric acid method (Liu et al., 2016). The total energy was calculated on the basis of the following equation: total energy (kJ) = 17 × (g crude protein + g total carbohydrate) + 37 × (g crude fat) (Liu et al., 2016).

### Amino acid analysis

The hydrolyzed amino acid compositions of the different samples were determined using a Thermo Fisher U3000 high-performance liquid chromatography system. Approximately 2 g of the samples was mixed with 16 mL of 6 M HCl in 20 mL hydrolysis tubes and subsequently vacuum degassed for 30 min. The tubes were then sealed with N_2_ and hydrolyzed at 110°C for 22–24 h. When cooled, the mixtures were transferred to 50 mL volumetric flasks, and their volumes were fixed to the scale. The hydrolysate (1 mL) of each sample was vacuum dried, and the precipitate was dissolved with 1 mL of 0.02 M HCl. Afterwards, 500 μL of the solution was mixed with 250 μL of 1 M triethylamine acetonitrile solution, and 250 μL of 0.1 M phenyl isothiocyanate acetonitrile solution. The obtained mixture was subsequently left at room temperature for 1 h. The mixture was added with 2 mL of *n*-hexane, intensely shaken and allowed to stand for 10 min. The resulting solution was filtered with a 0.22 μm aqueous filter membrane for analysis.

### Statistical analysis

Statistical analyses were performed using SPSS version 18.0. One-way ANOVA was conducted to analyse significant differences among multiple samples at a 0.05 level.

## Results

### Contents of mineral elements

**Figure 1:**
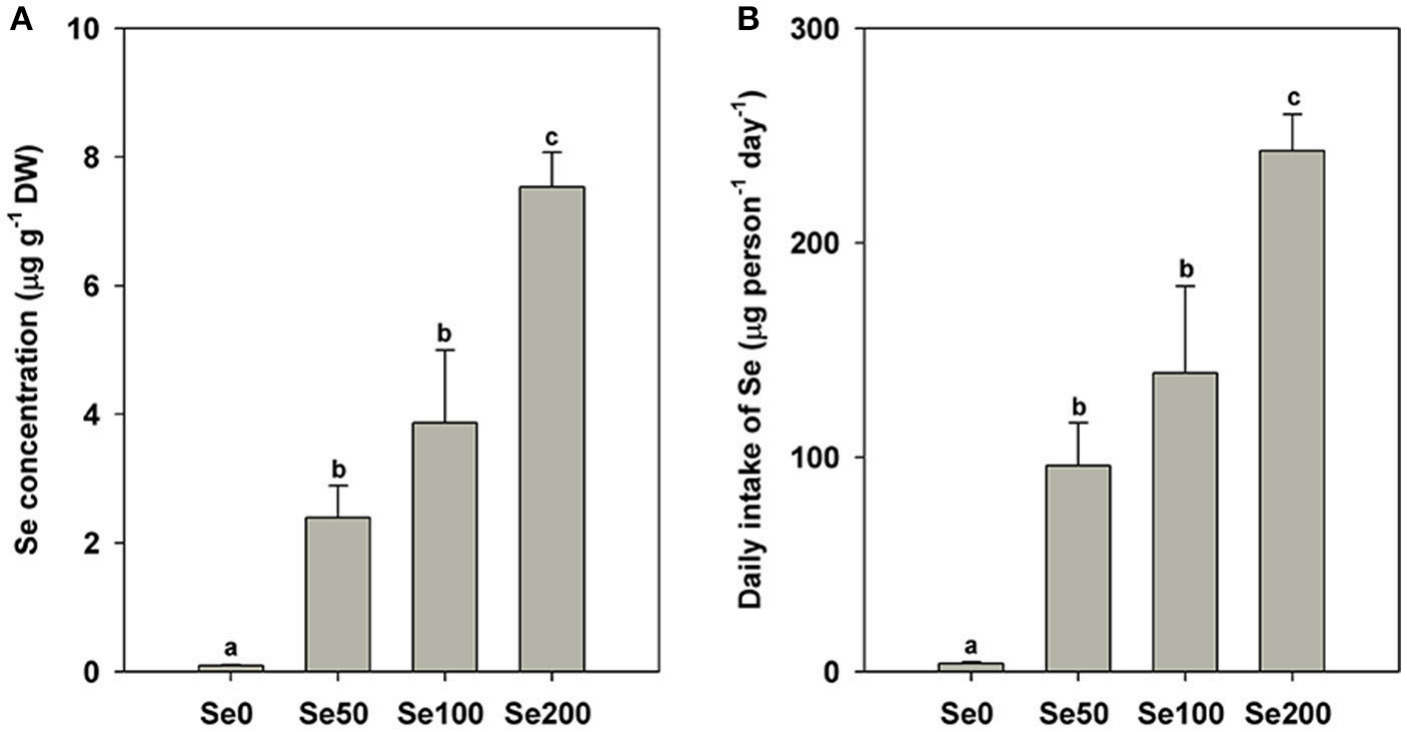
Se contents in different samples **(A)** and estimated daily intake of Se for adults from different samples **(B)**. Bars are means ± standard deviation. Bars labeled with different letters are significantly different at *P* < 0.05.

The contents of the mineral elements expressed on a DW basis in different samples are shown in Figure 1A and Table 2. The results of Se concentrations indicated that the foliar application of selenite could significantly increase Se accumulation in turnip fleshy roots (0.09 μg g^−1^ DW in the control samples and 2.39–7.54 μg g^−1^ DW in the Se-treated samples; *P* < 0.05; Figure 1A). On the basis of the Se concentrations in the fleshy roots, we estimated the following daily intake of Se for adults by feeding the turnip samples treated with the respective foliar applications of 0, 50, 100, and 200 mg L^−1^ Se (IV): 3.89, 96.08, 139.23, and 242.77 μg (Figure 1B).

**Table 2:** Contents of mineral elements in different samples.

**Mineral elements**		**Samples**			
		**Se0**	**Se50**	**Se100**	**Se200**
Macroelements (mg g^−1^ DW)	Ca	4.36 ± 0.64a	7.27 ± 1.69a	6.00 ± 1.79a	6.86 ± 1.04a
	K	10.72 ± 2.23a	8.45 ± 0.40a	8.60 ± 0.75a	11.48 ± 0.58a
	Mg	0.94 ± 0.24a	1.11 ± 0.08a	1.24 ± 0.08a	2.12 ± 0.61b
	P	1.49 ± 0.05a	2.05 ± 0.18a	3.17 ± 0.49b	2.36 ± 0.48a
Microelements (μg g^−1^ DW)	Fe	164.34 ± 24.11ab	125.01 ± 5.44a	205.51 ± 39.62b	137.41 ± 7.77a
	Zn	36.23 ± 3.48a	35.61 ± 1.32a	57.99 ± 3.26b	50.24 ± 5.87b
	Mn	10.02 ± 1.35ab	8.20 ± 0.35a	14.26 ± 0.78c	12.66 ± 2.89bc
	Cu	2.01 ± 0.14a	2.07 ± 0.14a	3.50 ± 0.11c	2.79 ± 0.45b

*Values are means ± standard deviation. Means within a row with different letters are significantly different at P < 0.05*.

To assess the effects of Se biofortification on the nutrients of turnip, we detected other essential mineral elements in the samples. In this study, the foliar application of Se (IV) did not elicit significant effects on the uptake of the macroelement Ca (4.36–7.27 mg g^−1^ DW) and K (8.45–11.48 mg g^−1^ DW) (Table 2). The Mg content in turnip significantly increased at 200 mg L^−1^ Se (IV) treatment (2.12 mg g^−1^ DW) (Table 2). The Se (IV) treatment at 100 mg L^−1^ increased the P content (3.17 mg g^−1^ DW) in the turnip fleshy roots, but a higher Se (IV) concentration (200 mg L^−1^) did not produce the same stimulus effect (Table 2). By comparison, the Se (IV) treatment further affected the uptake of microelements (Table 2). Furthermore, 100 mg L^−1^ Se (IV) treatment significantly improved the contents of Fe, Zn, Mn, and Cu in the turnip fleshy roots (*P* < 0.05) compared with those of the control samples, whereas 50 mg L^−1^ Se (IV) treatment had no such effects (Table 2). The contents of all of the four microelements decreased at 200 mg L^−1^ Se (IV) compared with those at 100 mg L^−1^ Se (IV) (*P* < 0.05). However, the contents of Zn and Cu remained higher than those of the control samples (*P* < 0.05; Table 2).

### Contents of macronutrients

The macronutrients and the total energy in different samples are presented in Table 3. The determined contents in a descending order were total carbohydrate (59.47–71.63 g/100 g DW), crude protein (5.95–9.35 g/100 g DW), and crude fat (5.90–7.76 g/100 g DW). The total energy (1414–1537 kJ/100 g DW) was also estimated. The crude protein content was significantly higher at 100 and 200 mg L^−1^ Se (IV) treatment (increased by 31.09 and 57.14%, respectively) than at the control concentration (Table 3). By contrast, Se (IV) treatment did not significantly affect the changes in the contents of total carbohydrate and crude fat and the amount of total energy (Table 3).

**Table 3:** Macronutrients (g/100 g DW) and total energy (kJ/100 g DW) in different samples.

**Sample**	**Components**			
	**Crude protein**	**Crude fat**	**Total carbohydrate**	**Total energy**
Se0	5.95 ± 0.13a	5.90 ± 0.92a	71.63 ± 5.66a	1537 ± 66a
Se50	6.71 ± 0.65ab	6.22 ± 2.02a	62.93 ± 15.84a	1414 ± 220a
Se100	7.80 ± 0.15b	7.76 ± 1.27a	62.80 ± 8.40a	1487 ± 192a
Se200	9.35 ± 1.15c	7.03 ± 0.87a	59.47 ± 3.24a	1430 ± 61a

*Values are means ± standard deviation. Means within a column with different letters are significantly different at P < 0.05*.

### Contents of hydrolyzed amino acids

The changes in the hydrolyzed amino acids (17 amino acids) in the turnip fleshy roots that were treated with different Se concentrations are presented in Table 4. The results showed that the foliar application of 50–200 mg L^−1^ Se (IV) did not change the contents of glutamate (3.67–5.89 g kg^−1^ DW), leucine (5.79–7.46 g kg^−1^ DW), and phenylalanine (4.19–4.60 g kg^−1^ DW) in turnip (Table 4). The contents of the 13 amino acids were improved by Se (IV) application at various levels. The contents of cystine, alanine, proline and lysine significantly increased when Se (IV) reached 100 and 200 mg L^−1^ (*P* < 0.05). By comparison, the contents of aspartic acid, glycine, histidine, arginine, threonine, tyrosine, valine, methionine, and isoleucine were not markedly induced until 200 mg L^−1^ Se (IV) was applied (*P* < 0.05; Table 4). The content of serine began to significantly increase at 50 mg L^−1^ Se (IV) (*P* < 0.05; Table 4). Overall, the contents of the total amino acids increased as the Se treatment concentrations increased (Table 4).

**Table 4:** Contents of hydrolyzed amino acids (g kg^−1^ DW) in different samples.

**Amino acids**	**Samples**			
	**Se0**	**Se50**	**Se100**	**Se200**
Aspartic acid	2.20 ± 0.38a	2.58 ± 0.26a	2.91 ± 0.25ab	3.71 ± 0.43b
Glutamate	3.67 ± 0.63a	5.19 ± 0.18a	5.27 ± 1.41a	5.89 ± 0.98a
Cystine	7.21 ± 0.13a	7.57 ± 0.09a	8.43 ± 0.29b	9.68 ± 0.43c
Serine	2.28 ± 0.13a	2.64 ± 0.13b	2.84 ± 0.20b	3.43 ± 0.11c
Glycine	1.59 ± 0.08a	1.92 ± 0.33a	2.33 ± 0.34ab	2.93 ± 0.48b
Histidine	1.84 ± 0.08a	1.98 ± 0.15a	2.11 ± 0.19a	2.68 ± 0.11b
Arginine	2.31 ± 0.26a	2.78 ± 0.26ab	3.48 ± 0.89ab	3.89 ± 0.55b
Threonine	1.36 ± 0.13a	1.72 ± 0.17a	1.76 ± 0.20a	2.21 ± 0.22b
Alanine	2.23 ± 0.17a	2.64 ± 0.18ab	3.00 ± 0.29b	3.21 ± 0.28b
Proline	1.50 ± 0.08a	1.75 ± 0.13ab	1.98 ± 0.06bc	2.25 ± 0.19c
Tyrosine	1.42 ± 0.08a	1.66 ± 0.13ab	1.66 ± 0.11ab	2.00 ± 0.34b
Valine	1.92 ± 0.17a	2.26 ± 0.22ab	2.72 ± 0.59ab	2.86 ± 0.22b
Methionine	5.29 ± 0.13a	5.53 ± 0.13a	5.94 ± 0.25ab	6.36 ± 0.18b
Isoleucine	2.37 ± 0.21a	2.75 ± 0.26a	2.88 ± 0.35a	3.46 ± 0.16b
Leucine	5.79 ± 0.05a	5.99 ± 0.28a	5.43 ± 1.50a	7.46 ± 0.55a
Phenylalanine	4.56 ± 1.17a	4.19 ± 1.15a	4.60 ± 1.09a	4.39 ± 0.21a
Lysine	3.62 ± 0.13a	4.01 ± 0.26ab	4.54 ± 0.53bc	5.07 ± 0.12c
Total amino acids	51.14 ± 1.36a	57.17 ± 1.45ab	61.88 ± 4.29b	71.48 ± 2.92c

*Values are means ± standard deviation. Means within a row with different letters are significantly different at P < 0.05*.

## Discussion

### Effects of Se (IV) on the contents of mineral elements

Consistent with the results from our previous study (Li et al., 2018), the foliar application of Se (IV) could significantly increase Se accumulation in the turnip fleshy roots. On the basis of the Se concentrations in the fleshy roots, we estimated the daily intake of Se for adults according to the formula used by Parveen et al. (2015). The daily Se intake from the turnip treated with the foliar application of 50 and 100 mg L^−1^ Se (IV) was in accordance with the dietary allowance of 55–200 μg Se day^−1^ for adults recommended by WHO (Wu et al., 2015). However, in our previous study, the daily Se intake from turnip treated with similar methods reaches 239 and 340 μg (Li et al., 2018). These values did not exceed the tolerable upper intake of 400 μg Se day^−1^ for adults as suggested by the Institute of Medicine (USA) (White, 2016), indicating that the foliar application of selenite should be an advisable method to consider turnip as Se-enriched food by biofortification. However, Se concentrations should be further validated according to actual environmental conditions, especially soil properties.

Interactions between Se uptake and other mineral elements have been studied in many plants, but some studies have reported ambiguous conclusions on these interactions. Some reports have supported our results that suitable Se concentrations could improve the uptake of various mineral elements, such as Mg, P, Fe, Zn, Mn, and Cu. Arvy (1992) considered that Se content is positively correlated with Mn, Zn, cobalt (Co), P, and molybdenum (Mo) concentrations in plants. Similarly, Chen and Liu (1996) reported that Se within the physiological concentration range can improve the uptake of P, K, Ca, Mg, S, Fe, Mn, Cu, Zn, and Mo in plants. Hu et al. (2015) also showed that Mn, Zn, Cu, Ni, and Co in the roots of Danshen are high when Se (Na_2_SeO_4_) is added. However, some of these studies have demonstrated that Se uptake can subsequently decrease the concentrations of other elements. For example, Arvy (1992) reported that Se content is negatively correlated with Fe, aluminum, and arsenic concentrations in plants. Singh and Singh (1978) also found that Se application reduces Fe, Mn, and Zn concentrations. Thus far, the interaction mechanism between Se uptake and other mineral elements is undefined. Se-induced decrease in the concentrations of some other elements may be attributed to ion antagonism. Fu et al. (2011) found that low Se (selenate or selenite) concentrations can improve the uptake of N, P, K, S, Mg, and Zn, whereas high Se concentrations inhibit the uptake of these elements in pak choi. In addition, some environmental factors may regulate the antagonism among different microelements. Singh and Singh (1978) found that increased Se levels reduce Zn and Cu contents in plants with a low P level, whereas Se application significantly increases Zn, Cu, and Mn contents when P fertilizer is applied. Thus, the effects of Se on the uptake of other mineral elements possibly depend on Se concentrations and soil conditions. However, our results provided evidence that the foliar application of Se (IV) could help improve mineral nutrient contents in turnip.

### Effects of Se (IV) on the contents of macronutrients

Our results showed that Se could improve protein synthesis in turnip, and similar results have been reported in several mushroom or plant species. Zhao et al. (2004) reported that Se can enhance protein content in *Ganoderma lucidum*. Yin et al. (2015) found that the crude protein content in potato increases by 4.87–5.44% after Se (0.379 kg hm^−2^) is applied. Nawaz et al. (2016) found that Se foliar spray (40 mg L^−1^) increases crude protein content by 47% in maize plants under drought stress conditions. These results indicated that Se affects N metabolism in mushrooms and plants. In contrast to our results, previous findings showed that Se can improve the contents of total carbohydrate, polysaccharide, or soluble sugar in mushrooms or plants (Shang et al., 1998; Zhao et al., 2004; Wang et al., 2006; Yin et al., 2015). For example, Shang et al. (1998) found that Se application can increase the total carbohydrate content in lettuce, and similar results are observed in the fleshy root of carrot (Wang et al., 2006). Related studies have shown that Se application positively affects the production of some organic nutrients, including carotene, crude fiber, vitamin C, and amino acids (Shang et al., 1998; Zhao et al., 2004; Wang et al., 2006; Yin et al., 2015). However, few studies have reported that Se can change the contents of total carbohydrate or crude fat. Du et al. (2004) revealed that 0.60 and 3.00 mg kg^−1^ of soil Se fertilizer significantly increase the content of crude fat in eggplant. Overall, our results were consistent with existing knowledge that Se can improve organic nutrients in plants. The Se-induced enhancement of organic nutrients may be closely related to Se assimilation and metabolism in plants, but the exact mechanism by which Se improves organic nutrition remains unclear.

### Effects of Se (IV) on the contents of hydrolysis amino acids

In this study, Se uptake elicited various positive effects on the synthesis of different amino acids, and the results were consistent with the change in crude protein. Previous studies indicated that Se can improve the contents of amino acids in some mushroom and plant species. Zhao et al. (2004) showed that moderate amounts of Se added in a culture medium can increase the contents of total amino acids and most components, except cystine in *G. lucidum*. Similar results have also been reported in two other studies (Niu et al., 1998; Wang et al., 2001). Du et al. (2004) found that soil Se fertilizer (0.60 and 3.00 mg kg^−1^) significantly improves the contents of total essential amino acids but does not obviously influence total amino acids in eggplant. However, they also observed that 0.15 mg kg^−1^ of soil Se fertilizer negatively affects the contents of amino acids in eggplant. In addition, Nawaz et al. (2016) demonstrated that 40 mg L^−1^ Se application enhanced the accumulation of total free amino acids (40%) in water-stressed maize plants. Thus, our results were partly consistent with previous findings, indicating that the effects of Se on amino acid synthesis were related to Se treatment concentrations and environmental conditions for different species. Se-induced changes in S and N metabolism in plants may be an important factor causing an increase in amino acid contents (Malagoli et al., 2015). For example, the contents of methionine and cystine can be improved to synthesize selenomethionine and selenocystine by inorganic Se because these amino acids contain S (Du et al., 2004). This conclusion is supported by the detected Se-methyl-SeCys in foliar selenate-treated radish (Schiavon et al., 2016) because the metabolism of inorganic Se in plants is usually carried out via the S metabolism pathway (Singh and Singh, 1979).

Among the Se-induced amino acids in our study, threonine, valine, lysine, methionine, histidine, and isoleucine are essential amino acids for humans (Galili et al., 2016). In addition, alanine, glycine, serine, and proline are considered as sweeteners, whereas aspartic acid exhibits umami characteristics (Ma et al., 2016). Thus, Se (IV) application for turnip might help improve amino acid nutrition and textures. The promotional effects of Se on amino acids were also observed at 50 and 100 mg L^−1^ Se (IV) (Table 4), which are the recommended concentrations for Se biofortification in turnip.

## Conclusion

This study demonstrated that the foliar application of selenite on turnip could significantly increase the Se content in turnip fleshy roots. Se (IV) application positively affected the uptake of several other mineral elements, such as Mg, P, Fe, Zn, Mn, and Cu. The foliar application of Se (IV) could improve the synthesis of proteins and multiple amino acids, including several essential amino acids, in turnip. Although Se (IV) treatment did not affect the contents of Ca, K, crude fat, and total carbohydrate and the amount of total energy, the results indicated that the foliar application of Se could enhance Se biofortification in turnip and promote its nutritional value. We recommended 50–100 mg L^−1^ Se (IV) treatment for foliar applications on turnip based on the daily intake of Se for adults. These Se treatment concentrations elicited favorable effects on the nutrient components of turnip. We also considered that the effects of Se on the nutrient components of plants are closely related to the soil environment. This study presented the feasibility and benefits of Se biofortification in Chinese turnip.

## Author contributions

YY and XL conceived and designed the experiments. XL and BL performed the experiments. XL analyzed the data and wrote the manuscript.

### Conflict of interest statement

The authors declare that the research was conducted in the absence of any commercial or financial relationships that could be construed as a potential conflict of interest.
